# “This is not what I imagined motherhood would look like”: pregnancy, postpartum, and parenting during COVID-19 – a qualitative analysis of the first year since birth

**DOI:** 10.1186/s12884-023-05872-3

**Published:** 2023-08-11

**Authors:** Lisette Saleh, Sharon Canclini, Cheryl Mathison, Shanna Combs, Beth Dickerson

**Affiliations:** 1 Texas Christian University, Fort Worth, USA; 2 Anne Marion Burnett School of Medicine at Texas Christian University, Fort Worth, USA; 3 Texas Health Resources, Fort Worth, USA

**Keywords:** COVID-19, Birth, Parenting, Motherhood, Postpartum, Mental health

## Abstract

**Background:**

Childbearing is one of the most emotional and transformative events in a woman’s life. This study aims to explore the impact COVID-19 had on childbirth, postpartum, and the first year since giving birth.

**Methods:**

This was a qualitative study using data previously collected for a larger study of women who had given birth during the COVID-19 pandemic in the United States. The findings presented here are from an analysis of a subset of open-ended questions. Sixty-six participants completed questions about how COVID-19 affected childbearing and postpartum experiences. Data was analyzed using inductive thematic analysis.

**Results:**

Thematic analysis of the data identified five major themes and several subthemes, including: (1) amplification of new mother typical emotions (positive emotions and negative emotions), (2) financial impact on mothers and their families, (3) persistent impact of COVID-19, (4) new mom paradigm crash (first time mothers and experienced mothers faced different issues such as lack of education and support, adding a layer to the day-to-day, and negotiating time with others) and (5) validating the importance of maternal health. On the whole, participants were overwhelmed, isolated, and did not have enough physical and emotional support. There was a lack of supportive maternal healthcare both in the short-term and long-term, with an emphasis on poor postpartum support.

**Conclusions:**

This study supports previous findings that women who gave birth and entered motherhood during the COVID-19 pandemic were impacted in many ways. These findings contribute to the understanding of women’s experiences not just in the immediate postpartum period, but in their daily lives one year after childbirth. The results highlight that our nation’s traditional maternal healthcare model may be insufficient, especially when facing a national crisis. Strain placed on the healthcare system by COVID-19 impacted both the physical and mental health of mothers who were often left with inadequate care, education, and support. Our findings point to the need for more supportive maternal health both during childbirth and postpartum.

## Background

In 2020, a novel severe acute respiratory syndrome coronavirus 2 (SARS-CoV-2), also known as coronavirus disease-2019 (COVID-19), was recognized as a global pandemic. By mid-March 2020, COVID-19 was declared a national emergency in the United States (US), creating a need for change in healthcare provision and lifestyle for families across the nation [1]. In the beginning, little was known about the virus. Healthcare systems, administrators, and providers had to determine how best to provide care in the face of a lack of scientific knowledge regarding the short and long-term impact. While hospitals worked to shutter units considered non-essential, several areas of healthcare such as labor and delivery and mother/baby units were deemed necessary and therefore stayed open. At the time of this article, there have been over 1.1 million deaths in the United States due to COVID-19 [2], 306 of them were pregnant. Over 225,000 pregnant women have contracted COVID-19 to date [3].

The pandemic had a devastating impact on maternal health care systems across the nation with three out of five patients experiencing modified or cancelled appointments [4]. Additionally, patients experienced wide variation in hospital visitor policy [5, 6], alterations in their birth plan [5, 7, 8], unnecessary obstetric interventions [9–11], inadequate postpartum and breastfeeding support [8], and early discharge from the hospital [10, 12, 13]. Women were ushered out of hospitals quickly to avoid potential exposure [13], increasing their likelihood of experiencing a negative postpartum outcome [10]. Once home, they found themselves isolated with a newborn and inadequate postpartum care for their physical and mental health needs [14]. The abridged time they had to learn about postpartum and newborn care caused higher readmission rates for newborns, especially among first time mothers [12].

COVID-19 directed quarantines and social isolation caused women to forgo those people they rely on for help and instead navigate postpartum alone. Support offered by family and friends both physically and emotionally, termed instrumental support, is important to mothers as they recover [15, 16] and acts as a buffer for anxiety and depression [16]. Women who gave birth during the pandemic experienced higher depression rates [5, 17–22], more anxiety symptoms [5, 17–22], loneliness [19, 23], fear, a loss of social support [7, 14, 24], and higher hostility rates when compared to women who gave birth pre-pandemic [22]. Having low social support, which could include emotional, instrumental, informational, or appraisal support, is linked with higher anxiety and depression in postpartum women [16, 23, 25, 26].

Studies have shown that a negative birth experience, such as giving birth during a pandemic, can increase parenting stress [21, 27], negatively impact mother-infant bonding [21, 28], decrease self-efficacy as a mother [27, 29], and increase postpartum depression even up to 12 months postpartum [30]. Individuals who were parenting during COVID-19 experienced more worry, higher anxiety, and depression [31–33]. Specifically, mothers experienced higher stress and less time to focus on themselves. Parents surveyed during the pandemic were able to name a multitude of ways that COVID-19 had impacted their families’ emotional, physical, and financial health [32]. Frustration and fears related to the pandemic, caused higher depression rates among parents versus those without children during the pandemic. Furthermore, mothers were more likely to feel isolated and alone than fathers, with many expressing a sense of burnout due to an increase in maternal roles [31].

While healthcare leaders sought to understand the impact COVID-19 had physically on the pregnant population, women were struggling to access care and education. Women had inadequate resources, deficient or nonexistent education, constantly changing policies, and hard to understand logic related to the care they received. As we look to identify the long-term effects of COVID-19 on our lives, women who gave birth during the pandemic will have a deep-rooted psychological impact on their mental wellbeing and parenting experience. Through exploration of the experiences women had giving birth and parenting during the COVID-19 pandemic,, we can learn how to best improve infrastructures, resource availability, and meet the needs moms are experiencing. This study seeks to take current research a step further by evaluating the impact of COVID-19 not just at birth or immediate postpartum, but to include motherhood and the first year of life. This study used qualitative methods to explore and develop understanding of the effect of COVID-19 on: (1) childbirth, (2) postpartum, (3) motherhood, and (4) daily life one year from birth.

## Methods

### Design and setting

This study constituted analysis of a subset of data from a larger longitudinal study. Data was collected from January 2021 to December 2021. This study addressed data collected from participants who completed open-ended questions on two online Qualtrics surveys. The first one at the time of admission into the study and another one year from date of giving birth. Approval for this study was obtained from an Institutional Review Board. All participants were provided a copy of the digital consent to participate and gave electronic consent prior to participation due to COVID-19 restrictions, with the ability to withdraw from the study at any point.

### Sampling and recruitment

Women were eligible to participate if they had given birth to a singleton live neonate on or after March 13, 2020, were 18 years of age or older, and had no history of prior fetal losses greater than 20 weeks gestation. Purposeful sampling was used for recruitment, which was carried out through use of online social media postings and snowballing. Within the first 24 h of recruitment, 92 women reached out with interest in sharing their experiences, thereby validating the need for this study. In the larger study, 80 participants met eligibility and completed all data collection points over the course of a year. This subset analysis included only the data collected from participants’ (n = 66) answers to the open-ended questions and demographic information.

### Data collection

All participants completed two electronic surveys using Qualtrics to capture demographics and qualitative data. The first survey was comprised of demographic information, obstetric history, and COVID-19 policies. Demographic data collected included age, race, geographic location of birth, highest education, and household income. Obstetric history data included previous births, location of most recent birth, delivery method, COVID-19 testing, masking, and essential worker status. The second survey was sent one year from birth of baby and consisted of open-ended questions focused on COVID-19. The questions were:

1) Now that you have had time to reflect on your last birth, how did COVID-19 impact you during that time?

2) Has your recent experience (pregnancy/birth/motherhood during COVID-19) affected how you are caring for your newborn, other siblings?

3) How is COVID-19 impacting your life today? Interview questions were designed by a research team based upon their combined clinical experience. The surveys were content validated by a group of healthcare providers including a professional certified doula, a public health nurse, obstetrician/gynecologist, labor and delivery nurse, and a maternal-child nurse.

### Data analysis

Descriptive statistics were used to analyze demographic and childbirth information using IBM SPSS version 29. All responses to the open-ended questions contained in the Qualtrics survey were collated. Data analysis was completed using thematic analysis in order to establish themes important to the research questions.

Qualitative data was analyzed following Braun and Clarke’s six step thematic analysis [34]. All research team members familiarized themselves with the data and generated initial codes independently. The research team met to discuss findings and come to a consensus on codes. Once codes were identified, each research team member independently reviewed the data, while focusing on the codes to ensure no further codes were deemed important. Codes were then collated and the team identified themes and subthemes that emerged from the codes. Consensus on the themes was reached by the research team. Trustworthiness of data was attained through credibility, confirmability, transferability, and dependability [35]. Credibility was obtained through use of an interdisciplinary team with a variety of viewpoints and experiences allowing for research triangulation. Furthermore, the research team spent time familiarizing themselves with the data through prolonged engagement with the transcripts. Confirmability was obtained through bracketing of personal bias and each member keeping a research journal throughout the study. Journaling allowed for documentation of bias and ideas of each research team member [36]. Transferability was established through detailed information regarding participant recruitment, data collection, data analysis, and suggestions for future research. Dependability of the study data was met by keeping an audit trail of the process of the research study including the interview guide, recordings, transcripts, and process of data analysis.

## Results

Sixty-six women provided complete data for both surveys. As noted in Table 1, the average age of participants was 31 years (SD 3.892), with a range of 23 to 44 years. The majority of participants identified as White/Caucasian (83.3%, n = 55). The remaining participants identified as Hispanic/Latino (10.6%, n = 7), as Black/African American (4.5%, n = 3), and identified as two or more races (1.5%, n = 1). Most participants had a college degree or higher in education (77.2%) (n = 51), and a household income of at least $80,000 (69.6%, n = 46). Furthermore, as shown in Table 1, the majority gave birth vaginally (81.8%, n = 54), in a hospital (92.4% n = 61), and were tested for COVID-19 when admitted (60.6%, n = 40), with only one testing positive for COVID-19.

**Table 1:** Demographics and Childbirth Information

Characteristics	Mean or n value	Range or percentage
**Age**	31	22–40
**Race**		
- White/Caucasian	55	83.3%
- Hispanic/Latino	7	10.6%
- Black/African American	3	4.5%
- ≥ Two races	1	1.5%
**Education**		
- High School Diploma	6	9.1%
- College Degree	6	9.1%
- Vocational Training	2	3.0%
- Bachelor’s Degree	19	28.8%
- Master’s Degree	21	31.8%
- Professional Degree	9	13.6%
- Doctorate Degree	2	3.0%
**Annual Household Income**		
- Under $20,000	1	1.5%
- $20,000-$40,000	6	9.1%
- $40,001 - $60,000	7	10.6%
- $60,001 - $80,000	6	9.1%
- $80,001 - $100,000	15	22.7%
- $100,001 - $120,000	9	13.6%
- $120,001 - over	22	33.3%
**Essential Worker**		
- No	36	54.5%
- Myself	12	18.2%
- Significant other	11	16.7%
- Both of us	7	10.6%
**Previous Cesarean section**		
- Yes	8	12.1%
- No	58	87.9%
**Birth Location**		
- Home birth	4	6.1%
- Hospital	61	92.4%
- Birthing center	1	1.5%
**Birth Method**		
- Vaginal Birth	54	81.8%
- Cesarean Birth	12	18.2%
**Tested for COVID-19**		
- Yes	40	60.6%
- No	26	39.4%
**Mask worn during birth**		
- Yes	32	48.5%
- No	34	51.5%

Thematic analysis of the data identified five major themes, exemplars from the surveys were taken verbatim to support the themes.

### Theme 1: amplification of new mother typical emotions

Women who gave birth during the pandemic recognized that the situation led to an amplification of new mother typical emotions. Generally, many postpartum women express feelings of fear, anxiety, exhaustion and of being overwhelmed. While these emotional states are the expected norm after giving birth, childbirth during a pandemic meant women spent their postpartum time at home isolated with their newborn. Therefore, there was an amplification of typical emotions that would usually be assimilated through interactions with other postpartum women during mother’s day out groups or social gatherings. Due to the lockdown, women had few ways to normalize their feelings through debriefing during social groupings. Most participants experienced a loss of physical and emotional support from family and friends, leaving them to rely on their spouse or the family living in the same home. They expressed a need for help navigating the common postpartum emotional highs and lows. Furthermore, with education and healthcare access interrupted or cancelled due to COVID-19, women were unable to assess if their individualized experiences and feelings were typical. Amplification of new mother typical emotions was illustrated with two subthemes: positive emotional experiences and negative emotional experiences.

#### Positive emotions

Some of the participants reported that COVID-19 caused them to reevaluate the importance of different aspects of their lives. They were able to find gratitude for the special time they had with their newborn and family.

“It also provided me and my spouse extra time with our baby that we wouldn’t have had outside of these circumstances.” (P40).

Other women found strength in themselves despite isolation and identified their capabilities as a new mother. Lacking outside support people or access to resources, women gained a deeper insight into what they were capable of doing for their newborn and family.

“I wasn’t nearly as prepared to have him as I would have liked (no classes or shopping for his nursey in person), but it taught me that babies don’t need as much as I was being sold. It taught me that moms can do anything, even in isolation.” (P35).

One participant was empowered to utilize her role as a postpartum mother to help others, outside of family, through use of her breastmilk.

“I am now extending breastfeeding so that I can continue to produce breastmilk that has antibodies in it to give to my baby and other children/family if needed.” (P19).

When reflecting on their childbirth experience, significant others were often allowed to be present for birth and were the sole supportive person present once women were discharged home. Several participants mentioned enjoying the special time spent as a newly formed family. One participant expressed feeling like a stronger couple after childbirth, postpartum, and parenting together with only their spouse for comfort and strength.

“My husband and I did this together. It made us stronger.” (P39).

#### Negative emotions

The majority of women stated they had felt a heightened sense of negative emotions including fear, anxiety, isolation, and depression. Lack of conventional access to healthcare providers due to modified appointments resulted in women feeling greater fear and anxiety regarding COVID-19.

“I still feel very robbed of a “normal” birth experience and wish I didn’t have so much fear going into my labor and fourth trimester.” (P40).

“It made us even more isolated than we would have been with a newborn, and the anxiety was paralyzing. We still feel the effects.” (P46).

Another woman stated that “COVID has created an environment where fear is front and center. Trying to navigate that well, being smart but not controlled by fear, is tough.” (P16).

Lack of support in the home during postpartum and parenting lead to overly stretched mothers and significant others with no reinforcements to rely upon.

“Lots of worry. Loss of attention on myself and my child, which led to feelings of low self-worth and a whole list of other things related to it. Led to second guessing everything and keeping myself hermitted in a way.” (P26).

“I felt that my postpartum journey and breastfeeding journey were heavily affected by COVID and restrictions related to COVID. My postpartum depression and anxiety were heavily driven by isolation and separation from my support system.” (P9).

### Theme 2: financial impact on mothers and their families

COVID-19 impacted women and their families financially through lost jobs and wages, increased prices on household items, and costly testing and treatment for illness.

“I was laid off as a result of lack of funds due to COVID, my son’s daycare was closed for several weeks due to COVID outbreaks (therefore eliminating my husband and/or my ability to work), my son’s daycare was closed for three weeks” (P19).

Participants saw an increase in the need to focus on schooling and childcare in the home. COVID-19 created competing roles for mothers including caregiver, teacher of their other children, and diligent virtual employee. With a lack of childcare and increased fears of COVID-19 transmission, some women had to make a choice between parenting and working. Women were often fulfilling many roles, all while recovering from childbirth and bonding with their newborn.

“I’ve also had to navigate multiple roles merging: I’ve been an employee, a healing postpartum mom, a homeschool teacher, literally all at the same moment.” (P36).

Some of the women had already left the workplace to stay home with their children due to fears related to COVID-19 and childcare options. Others were actively considering the benefits and drawbacks to quitting their jobs.

“Yes, I had to step back from my full-time job to be home more with the kids.” (P32).

### Theme 3: prolonged impact of COVID-19

Participants were able to identify ways in which COVID-19 was still impacting their daily life one year after giving birth. As families moved forward with life, they had to create a new norm or routine that incorporated COVID-19 ideas into each day. Whether it was through a noticeable increase in hand washing or how they asked guests to prepare for a visit, COVID-19 impacted a wide range of decisions.

“It makes me hypervigilant about washing my hands and what my son touches and puts in his mouth.” (P46).

Several women also mentioned the politicization of COVID-19 as it related to personal philosophies and community policies. This politicization continued to be present even one year out. Despite the introduction of vaccines and an increased knowledge about the virus itself, the divisions within some families and friendships deepened.

“The vaccine debate, job in healthcare, kids back in school, all areas of my life are impacted by COVID.” (P55).

“It has become such a taboo topic in some circles, and you are judged so severely by certain groups for decisions you make regarding your own health.” (P66).

The long-term societal impact continues to be felt with daily interactions focused on COVID-19. Participants experienced a deluge of information on social media and news channels, as well as during discussions with family and friends.

“As a nurse and a new mom as well as being pregnant again, COVID is a beatdown. You can’t turn on the TV or go to work without hearing something else about it.” (P8).

Mothers had to incorporate the pandemic in many aspects when navigating parenthood and relationships. One participant plainly stated that this politicization has led to COVID-19 being added to the taboo dinner table discussion list, along with religion or politics.

“We can’t discuss certain topics around my in-laws because my mother-in-law thinks the vaccine is a conspiracy.” (P60)With the virus evolving and new variants continuing to emerge, women were thrust back into a place of fear and worry over the health and safety of their baby.

“After a year, with the surge of the delta variant, I still feel extraordinarily nervous in taking my baby out and involving myself in social activities, especially in the politically driven landscape, despite my vaccination.” (P9).

### Theme 4: New mom paradigm crash

After giving birth, women have an expected pattern of healing, support, celebration, and reorganizing of familial roles. However, this expected new mom paradigm did not occur for those who gave birth during the pandemic. Increase in stress and reduced access to information and support paralyzed these assumptions. Women were not able to move forward towards a comfortable routine and established norm. The new mom paradigm crash was illustrated with four subthemes: new mothers and experienced mothers faced different issues, lack of education and support, adding a layer to daily life, and negotiating time with others.

#### New mothers and experienced mothers faced different issues

If the participant was a first-time mother, she faced different dilemmas compared to a mother who had other children to care for. New mothers often felt robbed of their vision for childbirth and celebrating their newborn. Without experience to compare with, their pandemic experience stands alone viewed through a lens of disappointment. Participants spoke of missing out on celebrations for family and friends to meet the new family member and missing out on support they expected. Furthermore, they lacked access to prenatal and motherhood education that would have helped to ground their expectations and dampen fear and anxiety.

“I had such limited support that I was unprepared to cope well with labor and my husband was unprepared to help me cope.” (P29).

On the other hand, experienced mothers had previous knowledge to reflect on, thereby decreasing fear of the unknown as it related to childbirth and postpartum. However, these women faced the added circumstance of managing care of a newborn while also parenting other children. Their other children were in virtual school or unable to attend daycare due to COVID-19 policies. This required the mother to lose special time with their newborn and manage many more needs of others in the household. Having other children to care for caused extra stress for these mothers but it also acted as a protective barrier to the isolation that many first-time mothers experienced. Another issue that arose for experienced mothers was trying to find childcare for multiple children in order to attend healthcare appointments. This issue caused missed appointments and lack of follow-through for mental and physical care.

“I wasn’t very fearful of COVID itself; I was very frustrated to have to find childcare for my older kids in order to go to lactation appointments and address other postpartum needs.” (P57).

#### Lack of education and support

Participants complained about the lack of education on various subjects related to pregnancy, childbirth, and postpartum that were previously available prior to COVID-19. Mothers wanted to engage with healthcare providers or more experienced mothers to learn about what to expect. Moving to virtual prenatal visits and cancellation of childbirth education and hospital tours caused many mothers to arrive at the hospital for birth uneducated and fearful.

“All prenatal classes offered through my hospital/OB were canceled with no virtual.

option offered.” (P29).

There was no way for mothers, especially first-time mothers, to adequately prepare for what they were going to experience. Furthermore, many mothers planned on participating in groups with other mothers offered at their church or neighborhood. However, with all events and groups cancelled, mothers instead were left to figure out postpartum and parenting by themselves. If a woman chose to breastfeed, she did not have the option of a lactation consultant or another mother to visit and ask for advice. Many participants focused on the severe lack of postpartum education and support as a contributor to their negative feelings.

“Also didn’t have the usual support from moms’ groups because of COVID.” (P14).

#### Adding a layer to the day-to-day

Planning for the day as a parent already takes a great deal of time, patience, and understanding. However, during the pandemic participants incurred another layer added to their day-to-day thinking, the COVID-19 layer. COVID-19 directly influenced parenting decisions such as what childcare they were comfortable with, if they should stop working, and how they plan on socializing their child. Mothers realized that COVID-19 was now an integral part to all aspects of their daily life. Whether or not they could put their child in daycare had a domino effect on whether they continued working. If they chose to have a person come to their home for childcare, they had to worry about if that person brought COVID-19 with them.

“COVID has made me more aware of how many people are in rooms. How close people are around me. Not knowing how to greet someone in person because of handshakes.” (P52).

#### Negotiating time with others

While the mothers often spoke of their desires to spend time with family and friends, there was a COVID-19 philosophy to navigate. Each individual had variations of what they believed about the virus, the care they took to isolate, masking, and testing. Therefore, each mother had to first talk with those individuals to identify their philosophy and then make a decision as to whether they were comfortable socializing together.

“Many of our friends have set strict boundaries when it has come to COVID, so it’s not just our concerns that are preventing connection.” (P36).

Several participants mentioned having significant others or family members that have vastly different philosophies and how this put a strain on those relationships due to the discord. Changes to relationships caused an already limited support system to be weakened further.

“Many of our friends have set strict boundaries when it has come to COVID, so it’s not just our concerns that are preventing connection.” (P36).

### Theme 5: validating the importance of maternal health

Many mothers, especially first-time mothers, were reliant on healthcare visits and classes for information and encouragement. Instead, they experienced feelings of abandonment by the healthcare system due to COVID-19 policies and restrictions.

“Felt unsupported pretty much my whole pregnancy.” (P58).

During childbirth, women expressed an appreciation and understanding for the strain put on hospital staff, especially nurses. Due to the lack of education and guidance received prior to hospital admission, women found themselves relying more on already strained nursing staff to supplement their needs.

“I felt that had it not been for COVID, I may have received more help from the nursing staff. Maybe they could have spent more a moment or two helping me with coping techniques. I also would have also had access to prenatal classes through the hospital for a very small fee and been able to more adequately prepare for the marathon of labor.” (P29).

Mothers frequently recognized the maternal health system lacked the services needed to support them once they had given birth. They expressed that after childbirth they simply felt forgotten. Without postpartum services, they lacked access to mental health support and counseling, breastfeeding, or parenting advice.

“I was very frustrated to have to find childcare for my older kids in order to go to lactation appointments and address other postpartum needs.” (P9).

Prior to the pandemic, women who had given birth could learn from and lean on other mothers during social gatherings. However, due to restrictions regarding gatherings, these events did not happen. Therefore, there was an even greater need for a strong maternal healthcare system to be accessible. Several of the mothers specifically stated that their perinatal depression and/or anxiety worsened postpartum and was not properly addressed. Their mental health was assessed, but there was a lack of follow through due to modifications in scheduling and policies.

“COVID has made it harder to get help, from breastfeeding support to counseling. I also feel that COVID contributed to me feeling passed off at my 6-week checkup. We identified some concerns about PPD/PPA, I was handed a business card, and I have not heard from my [provider] since.” (P36).

## Discussion

The present study was conducted to explore how COVID-19 impacted the experiences of women who gave birth during the pandemic. The authors aimed to understand the impact the pandemic had on giving birth, postpartum, motherhood, and daily life one year from birth. Results of this study indicated that women overwhelmingly felt a negative impact of COVID-19 on multiple aspects from childbirth to the first year of life.

Findings are congruent with other studies that note maternal healthcare was heavily impacted by the pandemic. Women lacked access to education and support during pregnancy to prepare themselves for labor and birth. Several studies across various geographical locations and points along the COVID-19 timeline noted women’s concerns about the lack of childbirth education options [7, 37] and alterations or cancellations of prenatal visits [7, 37–39]. A scarcity of childbirth education options and modifications to prenatal visits potentially induced a heightened sense of fear and anxiety surrounding childbirth. The lack in options led to an inability to process fears and learn about unknowns. Similar to our findings, modifications to hospital policies included restrictions on support people present for birth [5, 7, 38], visitor policies [6, 37], and shorter hospital stays [10–12, 32, 37]. All of these modifications had ramifications on women’s childbirth experiences. Seefeld et al. [28] identified an association between fear of childbirth and a negative birth experience. Furthermore, a negative childbirth experience was significantly associated with a poor mother-child-bonding at 8 weeks postpartum. While this association does show to weaken over time, the importance of a positive childbirth experience for short and long-term consequences cannot be understated. Provider offices did not allow women to bring their children with them for their postpartum follow up visit. Finding appropriate childcare was often a limiting factor for attendance at appointments. Women experienced traumatic births from strict isolation, newborn removal after birth, exposure to COVID-19, or maternal/fetal complications. These women were often left with no one with whom they could process or debrief their experience. Overall, participants felt neglected by the healthcare system during their postpartum time.

Congruent to previous findings, participants experienced an amplification of typical new mother emotions due to the stressors involved with giving birth under COVID-19 policies. The amplification was extended with lack of overall support once they were home [14]. Some of these emotions relate to the timing of childbirth in relation to the COVID-19 timeline. Those who gave birth earlier in the pandemic had far less information and more chaotic policies for all healthcare interactions. In the early stages of COVID-19, there was a lack of consistency and lots of confusion about policies surrounding childbirth. Women were unclear about hospital policies including isolation and contact with those testing positive. Childbirth in the later phases of COVID-19 allowed for some relaxation on policies and isolation protocol due to a better understanding of the virus. Similar to our findings, common language was found throughout the literature related to their emotional state giving birth during a pandemic including fear, anxiety [17], depression [17, 18, 40, 41], isolation [42], and a feeling of being overwhelmed with managing a new work life balance [42]. Davenport et al. [42] found that during the pandemic women who were pregnant or within the first year postpartum had higher depression and anxiety scores when compared to women pre-pandemic. Once home, women lacked the social structure and support often afforded women who gave birth before the pandemic. This was especially seen with first time mothers who conveyed their loss over missed celebrations and typical new mother rituals such as family and friends meeting the newborn [14, 40, 43). In a study by Dib et al. [23], postpartum women who had contact with others through a mother-baby or breastfeeding support group had better mental health. Mothers need those interactions to debrief their birth experience and normalize feelings with other women experiencing the same moment in life.

While our study did not seek to understand coping, it is important to note that previous studies have identified methods that pregnant or postpartum women could utilize that does improve mental health. Some of our participants did cope better than others with the stressors imposed by COVID-19. While not expressly stated by participants, it is noted by authors that many that coped well had family living close by and/or a supportive significant other. Mothers with other children also tended to cope better as they did not have the childbirth fears, but instead could focus on COVID-19 specifically. In a study by Farewell et al. [26], women were able to find resilience and coping behaviors to manage the negative impact of COVID-19 on their mental health and wellbeing. To help relieve some of the negative emotional toll, women actively sought time outside in greenspace, healthy habits, being proactive in connecting with others [24], and physical activity [41]. Healthcare providers can work to help mothers identify and implement coping methods that are easily achieved and help mental health during times of crisis.

Similar to previous findings, while participants experienced negative emotions, they also found an appreciation for the required shift in priorities and family bonding [38, 43]. Several participants found inner strength and had a positive response to the focused family time during the days after they were discharged home. It is interesting to note that our participants did not mention their significant other often when reflecting on these influential times in their lives. The authors posit this is an outcome of the woman’s focus on their individual experience during childbirth and postpartum. Furthermore, when significant others were mentioned, it was in relation to the fact that they were ill prepared to be the woman’s only support during labor. There is a definite need to provide education to significant others so they are prepared if they become the primary provider of care and support. While empowerment is not a common theme in COVID-19 childbirth literature found to date, Rice et al. [11] identified a similar sense of empowerment after giving birth during the pandemic. Through lack of support and resources, women learn to be self-sufficient and gain insight into their individual abilities as a mother. Furthermore, some women who gave birth during the pandemic found gratitude for the quality time with their newborn and family [20, 40].

While many studies identified negative consequences from childbirth to six months postpartum, our participants overwhelmingly noted a prolonged impact one year later. While COVID-19 was no longer in the initial stages during this study, the threat of new variants caused continued worry. Additionally, an increase in gatherings caused heightened anxiety for some women. The never-ending feeling of worry, fear, or anxiety related to unknowns caused isolated mothers to further retreat. Echoing previous studies, several participants worried about the health of their newborn. Many looked to the future and the impact the pandemic would have on the lack of socialization and development of their child [43, 44].

The data we collected presented considerable differences in the experiences of women who had other children and those who were first time mothers. In a recent study, Critchlow et al. [44] found that women who had other children felt they had no reprieve at home and often met roadblocks in finding appropriate childcare. In our study, participants with multiple children mentioned issues related to childcare, such as fear related to transmission, required COVID-19 testing, all of which added stress to their daily schedule.

Information about financial impact on participants was not directly asked, however, multiple women mentioned the ways they felt that COVID-19 impacted them financially. Some study participants and their significant others faced layoffs. Many participants experienced shifts in their roles at home requiring more of their mental and physical presence away from work, even in a virtual setting. Mothers with multiple children discussed difficulty in finding childcare and paying for testing of their children to meet required COVID-19 policies. Our findings were consistent with those reported by Dib et al. [23], who found that some women expressed a negative impact of the pandemic on their ability to pay for housing, food, and essentials. An evaluation of the impact of COVID-19 on gender equality found that while typical recessions tend to impact men’s employment more, the pandemic will severely impact women’s employment [45]. Several factors play into this financial impact, including flexibility of work for telecommuting, division of childcare among parents, and critical occupation status [45]. Due to a typical inequal division of childcare work towards women, their work life often suffers with fewer available hours to work and a higher likelihood to transition from working to a stay-at-home mother [46].

Determination of protective mechanisms to improve birth and postpartum experiences for women during the pandemic has included midwifery led care as well as home birth. Women who decided to stay out of hospital were more likely to experience quality perinatal care as compared to those who gave birth in hospital [47]. These births allowed for a decrease in obstetric interventions and options for inclusion of all birth plan desires that were often hindered by hospital policies.

### Limitations

The data collected included experiences and perspectives of women who self-identified for participating in the study. While attempts were made to recruit from a diverse geographical location, the majority of participants were from the South. Furthermore, the participant group lacked diversity in line with current US census. With a predominately white, educated, and high household income, it was difficult to generalize the findings for all women with similar experiences. We did not include inclusive language or seek individuals who do not identify as cisgender female. This should be considered in future studies to explore their experiences of childbirth and postpartum. The authors recommended future studies that seek a diverse group of women to include age, race, gender identity, geographical location, education, and socioeconomic level. Other recommended studies include investigating differences experienced by women living in multi-generational homes or are part of cultures that are matriarchal and community focused. These women may have had a greater integrated support system versus women in nuclear or single-family homes. Another aspect of interest is the significant others’ experience or behaviors that were supportive for women during this time.

While the women spoke overwhelmingly positive for the nurses who cared for them, they also felt lost and unable to find the resources and help they wanted. Future research should focus on the experience of the maternity healthcare provider to determine if they had the knowledge regarding resources and ways to assist these women. Focus should be on how information was dispersed to nurses and how maternal health policies should change to improve the birth and postpartum experience. This study revealed more about the experiences and perceptions of mothers further out from childbirth yet still feeling the impact of COVID-19 on their daily life.

## **Conclusions**

These findings are important to inform maternal healthcare and support, specifically in the postpartum period. Healthcare professionals need to recognize that postpartum care does not stop at the one postpartum visit within the first month or two. Many women were not able to attend their appointments due to childcare or cancellations and were left to create solutions for finding information and support they lacked. There is a definite need for development of evidence-based and accessible prenatal and postpartum education and resources. Furthermore, there is a need for institutions to develop a plan for the next major crisis. Within the plan they should consider the fact that labor and delivery and mother/baby units will never shut down. These units serve an important and unique, albeit generally healthy, population that should not be viewed collectively with other units and acutely ill patients. Pregnancy is a not a disease state and therefore policies should be logical and take into account the specific needs of pregnant and postpartum women.

This study identified the large gap between postpartum maternal need and access to maternal healthcare. Healthcare providers worked to make the best of a difficult situation but were forced to conform to hospital policy. Those policies often were driven by a focus on the COVID-19 units and a desire to decrease transmission. Unfortunately, the long-term impact of isolation for mothers while laboring, removal of newborns from healthy women, and the lack of postpartum support will be felt for many years to come.

The pandemic set women back when considering the progress made related to working equity. Furthermore, the creation of imposed virtual working conditions did not allow for full recovery of the woman after childbirth. There is a need to support women and reimagine gender roles, include gender specific support, and improve healthcare policies. Maternal mental health has consistently been a concern of multiple national organizations and yet no headway has been made. COVID-19 exposed the generalized lack of mental health and wellness care not only in women’s health care settings, but in all of the American health care system. As we continue to tackle the increased maternal morbidity and mortality rates in the United States, one area of major focus should be on the much-needed recognition that the postpartum period is an equally important time. While women in our study had the added struggles related to COVID-19, it further highlighted the lack of support and infrastructure in the maternal healthcare system, especially in the postpartum period, that is so desperately needed.

## Data Availability

The datasets used and analyzed during the current student are available from the corresponding author on reasonable request.

## List of abbreviations

Covid-19: coronavirus disease − 2019
US: United States
PPD: Postpartum Depression
PPA: Postpartum Anxiety
